# Disinfection Potential of 980 nm Diode Laser and Hydrogen Peroxide (3%) in “Critical Probing Depths” Periodontal Pockets: Retrospective Study

**DOI:** 10.3390/life12030370

**Published:** 2022-03-03

**Authors:** Marwan El Mobadder, Samir Nammour, Melanie Namour, Amaury Namour, Kinga Grzech-Leśniak

**Affiliations:** 1 Dental Surgery Department, Wroclaw Medical University, 50-425 Wroclaw, Poland; kgl@periocare.pl; 2 Department of Dental Sciences, Faculty of Medicine, University of Liege, 4000 Liege, Belgium; s.namour@uliege.be (S.N.); melanienamour@gmail.com (M.N.); amaurynamour@gmail.com (A.N.)

**Keywords:** periodontitis, non-surgical treatment of periodontitis, periodontal pathogens, periodontal disease, biofilm

## Abstract

A successful treatment of periodontitis depends largely on the successful elimination of the periodontopathogens during non-surgical and surgical mechanical debridement. In this retrospective study, data collection was conducted from 2017 to 2021. The retrospective study included 128 patients with 128 sites of localized periodontitis with pocket depths > 5 mm. The included data were based on sites that received conventional mechanical debridement followed by different adjunctive approaches. In total, 30 patients did not receive any additional treatment (SRP group), 30 patients received SRP + 980 nm diode laser irradiation only (SRP + laser), 30 patients received SRP + 3% hydrogen peroxide irrigation (SRP + H_2_O_2_) only and 30 patients received a combined treatment of 3% hydrogen peroxide and 980 nm diode laser irradiation (SRP + H_2_O_2_ + laser). Total bacterial counts (TBC) in the periodontal pocket collected for all participants before treatment, immediately after treatment, 6 weeks after treatment, 12 weeks after treatment and 6 months after treatment were statistically analyzed and compared. When the laser was used, irradiation parameters were 10 μsec/pulse duration, 10 kHz, pick power of 10 W, average power of 1 W, irradiation time of one minute with inward and outward movements, and fiber diameter of 320 μm. The irradiation was repeated 3 times/pocket. When hydrogen peroxide was used, the irrigation was conducted for one minute and repeated 3 times. The maximum reduction in TBC was obtained when SRP was coupled with 3% H_2_O_2_ irrigation followed by 980 nm diode laser irradiation. After six months of follow-up, a significant reduction in TBC was obtained for the group of SRP + H_2_O_2_ + laser when compared to all the other groups, from 7.27 × 10^7^ before intervention to 3.21 × 10^7^ after six months. All three approaches to SRP showed a significant reduction in TBC immediately after treatment. Values were 3.52 × 10^7^, 4.01 × 10^6^, 9.58 × 10^6^, 1.98 × 10^6^ for SRP alone, SRP + diode, SRP + H_2_O_2_ and SRP + H_2_O_2_ + diode laser, respectively. At 6 months, we saw no significant difference between SRP + laser and SRP + H_2_O_2_ with 4.01 × 10^7^ and 4.32 × 10^7^, respectively. This retrospective study reveals that after SRP, irrigation with 3% hydrogen peroxide and irradiation with a 980 nm diode laser within specific treatment protocol can be used as an additional approach to conventional SRP to increase the disinfection of the periodontal pockets > 5 mm.

## 1. Introduction

Periodontitis is a multifactorial, biofilm-induced chronic inflammatory disease affecting and leading to a destruction of the periodontium and ultimately tooth loss [1,2]. Periodontitis has a major negative impact on the patient’s quality of life and has been well documented to be associated with systematic conditions such as cardiovascular disease, rheumatoid arthritis, diabetes mellitus, respiratory disease, pregnancy and others [2]. 

Although investigations continue, the exact mechanism of action of the periodontal pathologies, notably periodontitis and the transition from gingivitis to periodontitis, is still not fully understood [3,4,5]. Yet, it is agreed on today that the shift from stable gingivitis to periodontitis requires both dysbiotic microbiota and a susceptible host [6,7,8]. In fact, it is the interaction between dysbiotic microbiota and the host response that leads eventually to a complex inflammatory exchange including synergistic interactions of the microbiota for enhanced colonization, nutrient procurement and persistence in an inflammatory environment that promotes their adaptive fitness [6,7,8,9]. Therefore, a better disinfection after mechanical debridement of the supra- and subgingival calculus inside the periodontal pocket should theoretically result in a better perturbation of the dysbiotic environment essentially controlled by anaerobic periodontopathogens, principally *Porphyromonas Gingivalis*, *Aggregatibacter actinomycetemcomitans*, *Prevotella intermedia* and *Tannerella forsythia*. Hence, better disinfection is still considered as the standard of care to improve the periodontal tissue reparation [10,11,12,13,14]. On the other hand, proper oral hygiene is also still considered as gold standard for improved periodontal health. Studies reveal that in the majority of the cases a reduction of 32% to 37% of the plaque index with a total reduction in bleeding upon probing is seen in patients with proper oral hygiene [15].

While SRP can offer mechanical debridement by direct contact of both hard and soft tissue, the use of a light source such as a laser as an additional tool can offer a deeper penetration of the energy which may eradicate more pathogens [16]. In this context, one of the promising additional approaches used with non-surgical and surgical mechanical debridement is antimicrobial photodynamic therapy (aPDT) [16,17,18]. aPDT is a form of phototherapy involving light and a photosensitizing chemical substance to elicit phototoxicity. Among the used photosensitizers in periodontology are methylene blue, malachite green and rose bengal. aPDT includes two components: a photosensitizer and a light source. The light will activate the photosensitizer which will generate reactive oxygen species that are responsible for the antimicrobial property of the PDT [16,17,18]. However, hydrogen peroxide (H_2_O_2_), in opposition to classic antimicrobial photodynamic therapy (aPDT) is believed to offer higher bioavailability and deeper penetration in biofilms as well as in the scarce interference [16,17,18]. aPDT today presents a broad range of applications in odontology. For instance, aPDT was suggested as a promising approach for the management of leukoplakia and pre-neoplastic lesions of the oral cavity [16], for the management of oral complications due to cancer therapy [17], for the treatment of dentinal hypersensitivity associated to exposed dentin [18] and for the management of halitosis [19]. These are among the indications that can be found in the literature about the possible benefits of aPDT in dentistry.

The combination of a 980 nm diode laser that can present a high penetration and H_2_O_2_ can theoretically disinfect out of the range of root surface instrumentation when compared to mechanical debridement and might interact with the periodontal pathogens residing in depth of the periodontium [20,21,22,23,24,25,26,27,28]. However, there is still a need to assess the total bacterial count inside the periodontal pocket before and after different protocols combining the use of 3% H_2_O_2_ and a 980 nm diode laser in the critical probing depth of periodontal pockets and within a six-month follow-up.

Hence, the aim of this retrospective study is to assess the disinfection potential of different adjunctive methods after scaling and root planning (SRP) for the treatment of periodontitis stage III and IV (according to the new classification of the European Federation of Periodontology and the American Academy of Periodontology) on the periodontal pathogens: SRP alone, SRP + irrigation with 3% hydrogen peroxide, SRP + irradiation with a 980 nm diode laser and the combination of both. The null hypothesis was that there is no difference in the disinfection potential between SRP and any additional adjunctive approach.

## 2. Materials and Methods

### 2.1. Study Design

This is a retrospective study conducted by data collection of four different group of participants that underwent four different interventions as non-surgical treatments of periodontitis. Data collection was performed from 2017 to 2021. The total number of participants included in this retrospective study was 120. All participants received the gold standard non-surgical mechanical debridement consisting of oral hygiene instruction and scaling root planning (SRP) with an ultrasonic piezoelectric scaler (Piezosteril 6, Castellini, Cazzago San Martino BS, Italy). Three groups received different adjunctive approaches in addition to the non-surgical treatment. The 4 groups in the retrospective study were:

- Group SRP: scaling root planning only (*n* = 30; control group).
- Group SRP + laser: scaling root planning + irradiation of the concerned pocket with the 980 nm diode laser only (*n* = 30).
- Group SRP + H_2_O_2_: scaling root planning + irrigation of the concerned pocket with 3% hydrogen peroxide for one minute (*n* = 30).
- Group SRP + H_2_O_2_ + laser: scaling root planning + irrigation of the concerned pocket with 3% hydrogen peroxide and then irradiation inside the pocket with the 980 nm diode laser (*n* = 30).

Data from patients that received one of the mentioned treatments and having localized periodontitis stage IV (according to the new classification of the European Federation of Periodontology (EFP) and the American Academy of Periodontology (AAP)) were retrospectively collected. Therefore, localized periodontitis with a pocket > 5 mm was included. This study was conducted according to the Declaration of Helsinki. Our retrospective data collection does not legally need prior approval from an ethics committee.

### 2.2. Participants

In total, 120 periodontal pockets greater than 5 mm were included in this retrospective study. All participants signed a written informed consent form before their enrollment after a thorough explanation of the study’s requests and its possible benefits and complications.

#### 2.2.1. Inclusion Criteria

- Localized periodontal pocket with a probing depth > 5 mm and a clinical attachment loss of >3 mm (periodontitis stage III or IV).
- Motivated patients with adequate oral hygiene (plaque index < 30%).
- Patients that signed the written informed consent form.

#### 2.2.2. Exclusion Criteria

- Presence of tooth mobility.
- Patients having any systematic disease that is considered to be a contraindication for a non-surgical periodontal intervention.
- Patients with plaque index > 30%.
- Patients who are currently taking or took antibiotics, probiotics or any other adjunction within the last 6 months.
- Patients who are currently taking or took immuno-suppressants within the last 6 months.

### 2.3. Non-Surgical Periodontal Treatment (SRP)

All participants (*n* = 120) underwent oral hygiene instruction and motivation to enhance their oral hygiene. Afterwards, professional scaling root planing both using an ultrasonic piezoelectric scaler and manually took place. After SRP, periodontal interdental brushes were prescribed to be used on sites with periodontitis. The shape and size of the interdental brushes were chosen according to each patient. SRP was performed mechanically with an ultrasonic scaler (piezosteril 6, Castellini, Cazzago San Martino BS, Italy) and then manually with periodontal curettes (Universal and Gracey curettes). A 0.12% Chlorhexidine solution (Eludril pro, mouthwash, Pierre Fabre Oral Care, Paris, France) was applied to each of the periodontal pockets at the end of the treatment. When required, extensive or defective restorations were corrected and reshaped. At the end of the mechanical debridement, chlorhexidine 0.12% (Eludril pro, mouthwash, Pierre Fabre Oral Care, Paris, France) was prescribed for 7 days, to be used once a day for one minute of mouth rinsing.

### 2.4. Group Scaling Root Planning + Irradiation with Diode Laser (SRP + Laser)

For the SRP + laser group, the laser was used right after SRP. The use of the laser was made as follows:

- Insertion of diode laser fiber (Smart M, Lasotronix, Warsaw, Poland) into 1 mm from the depth of the pocket.
- Irradiation with the laser in an inward and outward movement parallel to the longitudinal axe of the tooth, in a contact mode with the junctional and sulcular epithelium, frequency of 10,000 Hz, 10 µs pulse duration, 10 W pick power, 1 W average power and speed of movement of ±1 mm/s. The energy density was 31.25 J/cm^2^ per second. The fiber diameter was 320 µm and the irradiation time was 1 min.
- Repetition of the same procedure 3 consecutive times.

### 2.5. Group Scaling Root Planning + Hydrogen Peroxide (SRP + H_2_O_2_)

This group underwent scaling root planning + irrigation with hydrogen peroxide (SRP + H_2_O_2_). Hydrogen peroxide was used after SRP as follows:

- Deposition of H_2_O_2_ diluted solution (3%) in the pocket using a syringe with a needle of 0.3 × 25 mm.
- Removal of the excess of H_2_O_2_ that will come out of the pocket.
- Wait for one minute.
- Repetition of the same protocol 3 consecutive times.

### 2.6. Group Scaling Root Planning + Hydrogen Peroxide + Laser Irradiation (SRP + H_2_O_2_ + Laser)

For this group, the protocol after SRP was as follows:

- Deposition of H_2_O_2_ diluted solution (3%) in the pocket using a syringe with a needle of 0.3 × 25 mm.
- Removal of the excess of H_2_O_2_ that will come out of the pocket.
- Wait for one minute before irradiation.
- Irradiation with the 980 nm diode laser with the exact protocol and parameters described in Section 2.4.

### 2.7. Bacteriological Study and Follow-Up

A T40/04 sterile paper point was inserted into each selected periodontal pocket for 30 s and used for DNA probe analysis. In total, 120 data points were collected from the periodontal pocket from 120 participants. The paper points were then stored inside the tube according to the manufacturer’s recommendations. This sampling was conducted before any treatment, immediately after treatment, and at 6 weeks, 12 weeks and 6 months of follow-up. Total bacterial count (TBC) was taken with a with biological molecular test (PetPlus; MIP Pharma^®,^ Blieskastel, Germany). Samples were sent and analyzed blindly. The total bacterial count was determined using the universal probe. The results were translated by MIP Pharma^®^ into millions of bacteria by arbitrarily deciding that one bacterium was equivalent to 10^4^ copies of ssrRNA. Microbiological analysis for the detection of the bacteria was performed with the real-time polymerase chain reaction method and bacterial DNA was extracted according to the instructions of the manufacturer for total bacteria count (TBC).

### 2.8. Statistical Analysis

For the statistical analysis, Sigma five^®^ software was used (GraphPad Prism 5, San Diego, CA, USA). Statistical significance was considered when the *p* value was <0.05. The confidence level of the study was proposed to be 99% with a *p* value < 0.001, which is a very high significance. Mean and standard deviation (Std) were calculated for each group. Smirnov and Kolmogorov tests were utilized to assess the normality tests. One-way ANOVA coupled with a Newman–Keuls multiple comparison test (post hoc test) were used.

## 3. Results

**Figure 1:**
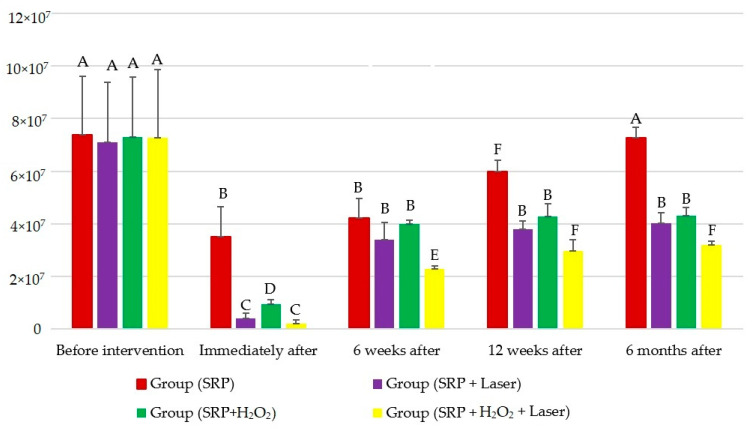
Mean and standard deviation of total bacterial count (TBC) for all groups. (Identical letters indicate the absence of a statistically significant difference, while the difference in letters indicates a statistically significant difference. *p*-value < 0.0001, all values are in CFU/mL. The vertical bars represent the mean values, and the vertical lines represent the standard deviation for each group. Values are in CFU/mL.)

After six months of follow-up, the most significant reduction in TBC was obtained for the group SRP + H_2_O_2_ + laser with a statistically significant reduction when compared to the other three groups. TBC of the group SRP + H_2_O_2_ + laser was 7.27 × 10^7^ CFU/mL before intervention and reduced to 3.21 × 10^7^ CFU/mL after six months of intervention (Table 1, Figure 1).

Therefore, the combination of 3% hydrogen peroxide with 980 diode lasers after SRP is the most effective procedure for the reduction in TBC in the periodontal pockets greater than 5 mm. In addition, the use of the 980 nm diode laser alone after SRP gave immediately after the intervention a significant reduction in TBC when compared to H_2_O_2_ alone after SRP or SRP alone. However, at six months of follow-up, there was no significant difference between SRP + laser and SRP + H_2_O_2_ with 4.01 × 10^7^ CFU/mL and 4.32 × 10^7^ CFU/mL, respectively. When compared to SRP alone, all three adjunctive approaches to SRP show a significant reduction in TBC immediately after treatment with values of 3.52 × 10^6^ CFU/mL, 4.01 × 10^6^, 9.58 × 10^6^ CFU/mL, 1.98 × 10^6^ CFU/mL for SRP alone, SRP + diode, SRP + H_2_O_2_ and SRP + H_2_O_2_ + diode laser, respectively, and after six months of follow-up with the values 4.01 × 10^7^ CFU/mL, 4.32 × 10^7^ CFU/mL and 3.21 × 10^7^ CFU/mL, respectively.

## 4. Discussion

In this study, the additional use of 3% hydrogen peroxide, the use of a 980 nm diode laser and their combination resulted in a significant reduction in TBC when compared to conventional SRP alone. This finding confirms that the 980 nm diode laser alone coupled with SRP, or the H_2_O_2_ alone coupled with SRP can lead to a better disinfection out of the range of root surface instrumentation achieved in the SRP. This may be due to the deeper microbial reduction obtained when additional approaches, such as the use of H_2_O_2_ or the laser are used, which will lead to a better killing of the bacteria residing in the depth of the periodontium and the non-periodontal mucosal surfaces. However, the highest disinfection of the pocket was obtained when hydrogen peroxide 3% was coupled with 980 nm diode laser after SRP. In fact, the combination of 3% hydrogen peroxide followed by the 980 nm diode laser gave a higher disinfection of the periodontal pocket. Values were higher than all groups and at all times of follow-up. Hence, the null hypothesis was rejected. Based on this retrospective study, it can be concluded that the combination of 3% H_2_O_2_ and a 980 nm diode laser as adjunction to SRP can be an effective therapeutic modality as adjuvant to non-surgical mechanical debridement in managing localized periodontitis with pockets >5 mm. This adjunctive application of hydrogen peroxide and diode laser led to an increased reduction in TBC and therefore can find its indication in localized periodontitis with deep pocket. The term “critical probing depths” used in this study was first introduced by Lindhe et al., referring to periodontal pockets that are greater than 5 mm [29]. These periodontal pockets are referred to as critical because a non-surgical mechanical debridment might not be sufficient to resolve the inflammation and stop the progression of periodontitis; therefore, a surgical intervention might be needed.

The findings in this retrospective study can be explained by several factors. The bactericidal effect speculated when the diode laser (980 nm) was used can be attributed to the known thermal and photo-disruptive effect of the laser irradiation that lead to sublethal damage of the bacteria [20,30,31,32,33]. In addition, the near-infrared light of the 980 nm diode laser can directly kill the pigmented bacteria (containing protoporphyrin IX), which increases the disinfection [30,31,32,33]. In fact, laser irradiation within adequate parameters and protocol is known to result in the disruption of the bacterial cell wall, disruption of the integrity of the bacteria, an accumulation of denatured proteins, and subsequently cell lysis and microbial death [20,34]. Therefore, this can explain why the SRP + diode laser group showed better disinfection when compared to SRP alone. On the other hand, H_2_O_2_ is a potent antimicrobial solution that covers a broad spectrum of actions, including activity against bacterial spores and viruses [21,22,23]. Its effectiveness has been attributed to the physical effect of the stream of H_2_O_2_ and to the bubbling of the oxygen as it is released from the peroxide [16,17,18]. Hydroxyl radicals are considered powerful oxidizing agents that can cause lethal oxidative injuries to some bacteria [21,22,23]. Therefore, this can explain why the SRP + H_2_O_2_ group showed significantly better disinfection potential when compared to SRP alone. However, when H_2_O_2_ was used with the diode laser, the disinfection potential was interestingly greater than when H_2_O_2_ was used alone or when the diode laser was used alone. This can be explained by the fact that H_2_O_2_ reacts slowly with biological materials in the absence of catalysts, such as the transition metal cations copper (CU^2+^), and that some biological properties and interactions between the pathogens can help resisting the antimicrobial effect of the H_2_O_2_ [35].

This speculated reduction in TBC obtained after combining 3% hydrogen peroxide and the 980 nm diode laser can be explained by the fact that the light of the laser can enhance the activity of H_2_O_2_ by prolonging the lethal oxidative damage of H_2_O_2_, which will result in the activation of a stronger bactericidal effect [16,17,18,20]. Therefore, the generation of reactive oxygen species following the collapse of the cavitation bubbles might be attributed to a much better antibacterial medium in the gingival tissue, which enhances the efficiency of the disinfection of the colonized root surfaces [16,17,18,20]. In addition, since deep pockets (>5 mm) with periodontitis were included, the bacteria involved were mainly gram-negative, found in the periodontal pocket. These gram-negative bacteria possess tough cell walls made of highly cross-linked murein, making them more resistant to laser irradiation alone [36,37,38,39,40,41,42]. Therefore, the combination of both laser and H_2_O_2_ might have overpassed these tough cell walls and made the anaerobic bacteria less resistant and more vulnerable to the disinfection process [40,41,42]. Moreover, it is reasonable to expect better disinfection potential when two different approaches are carried out in the same area because each agent (laser or hydrogen peroxide) will target different and specific pathogens and therefore their combination will result in a broader disinfection.

Numerous *in vitro*, *in vivo* and clinical studies investigated the bactericidal potential of additional approaches coupled to SRP treatments in patients with periodontitis [43,44,45,46,47]. For instance, Peihui Zou [43] demonstrated that reactive oxygen species delivered partially can kill *Porphyromonas gingivalis* and *Prevotella intermedia* by promoting the overproduction of ROS. Although promising, these results were more significant in planktonic state bacteria than in a biofilm state. In the biofilm state, the values were 0.20 log10 CFU/mL and 0.42 log10 CFU/mL for *Porphyromonas gingivalis* and *Prevotella intermedia*, respectively [43]. The results obtained by Peihui Zou et al. are similar to ours in the fact that reactive oxygen species can result in an effective bactericidal effect [43]. Similarly, Grzech-Lesniak showed in a randomized clinical and microbiological study that multiple applications of aPDT using toluidine blue 0.1% as a photosensitizer can be an effective additional approach to the SRP during the maintenance sessions for patients with periodontitis [44]. On the other hand, Bansal et al. [45] found that the use of a laser and chlorhexidine chip assures a significant reduction in TBC when compared to SRP alone. However, unlike our study, the follow-up after treatment was carried out only for 4 weeks [45]. In agreement with our findings, G. caccianiga et al. showed in a microbiological study that laser peroxide with hydrogen peroxide has a significant bactericidal effect on *Prevotella intermedia*, *Peptostreptococcus micros* and *Fusobacterium nucleatum* and that the best results were obtained when both hydrogen peroxide and a diode laser were combined on these three periodontopathogens [46]. However, this was a microbiological study with no follow-up period [46]. Similarly, to our study, Moritz et al. tested in a randomized study the effect of the 980 nm diode laser + SRP vs. hydrogen peroxide rinsing + SRP. Better bacterial reduction was obtained with SRP + diode laser compared to SRP + H_2_O_2_ alone according to Moritz et al.’s study [47]. Probiotics are also being suggested as an additional approach for the non-surgical treatment of periodontitis such as Akkermansia muciniphila [48]. These probiotics are showing promising results as an additional approach for SRP [48]. Moreover, Grzech-Lesniak showed that a combination treatment with Nd: YAG laser and 0.5% NaOCl or H_2_O_2_ results in more significant reduction in bacterial viability when compared with SRP as a monotherapy [36]. These findings in the present study are in accordance with the previous study [36].

However, Butera et al. failed to show an additional benefit in the total bacterial count reduction in *Aggregatibacter actinomycetemcomitans*, *Tannerella forsythia*, *Porphyromonas gingivalis* and *Treponema denticola* when the probiotic was introduced [49].

This clinical study assessed the TBC count reduction in deep periodontal pockets (>5 mm) with a follow-up of six months of SRP in addition to 980 diode lasers alone, hydrogen peroxide 3% alone, and their combination. As for the safety of the suggested treatment modalities, the 980 nm diode laser used in our study and within our protocol and parameters and the 3% hydrogen peroxide irrigation in the pocket depth were both confirmed to be safe in a plethora of studies. However, this retrospective study presents several limitations. One of the limitations is the absence of an assessment of the periodontal clinical parameters such as pocket depth, clinical attachment level and periodontal recession. Moreover, a one-year follow-up after treatment could have given more information on the progression of the total bacterial count. In addition, further studies are invited with a focus not only on the total bacterial count but also on the main periodontopathogens: *P. gingivalis*, *Aggregatibacter actinomycetemcomitans*, *Tannerella Forsythia* and *Prevotella intermedia*. Hence, we invite further studies to apply similar protocol and assess the variation in the clinical periodontal parameters with a longer period of follow-up. Hence, we invite further studies to apply the exact same protocol and assess the variation in the clinical periodontal parameters with a longer period of follow-up.

## 5. Conclusions

In conclusion, the retrospective study confirms that after conventional non-surgical mechanical debridement, irrigation with 3% hydrogen peroxide followed by irradiation with 980 nm diode laser under our specific irradiation conditions can provide a significant reduction in the total bacterial count in the periodontal pockets that are greater than 5 mm and within a follow-up of six months.

## Figures and Tables

**Table 1:** Mean and standard deviation of total bacterial count (TBC) for all groups.

	Group SRP		Group SRP + Laser		Group SRP + H_2_O_2_		Group SRP + H_2_O_2_ + Laser	
	Mean	STD	Mean	Std	Mean	Std	Mean	Std
Before intervention	7.40 × 10^7 A^	2.22 × 10^7^	7.10 × 10^7^ ^A^	2.28 × 10^7^	7.29 × 10^7 A^	2.28 × 10^7^	7.27 × 10^7 A^	2.60 × 10^7^
Immediately after	3.52 × 10^7 B^	1.14 × 10^7^	4.01 × 10^6 C^	1.93 × 10^6^	9.58 × 10^6 D^	1.46 × 10^6^	1.98 × 10^6 E^	1.42 × 10^6^
6 weeks after	4.21 × 10^7 B^	7.43 × 10^6^	3.41 × 10^7 B^	6.41 × 10^6^	4.00 × 10^7 B^	1.26 × 10^6^	2.28 × 10^7 E^	1.14 × 10^6^
12 weeks after	5.99 × 10^7 F^	4.31 × 10^6^	3.79 × 10^7 B^	3.20 × 10^6^	4.28 × 10^7 B^	4.80 × 10^6^	2.98 × 10^7 F^	4.13 × 10^6^
6 months after	7.26 × 10^7 A^	4.11 × 10^6^	4.01 × 10^7 B^	4.10 × 10^6^	4.32 × 10^7 B^	3.10 × 10^6^	3.21 × 10^7 F^	1.24 × 10^6^

Identical letters indicate the absence of a statistically significant difference, while the difference in letters indicates a statistically significant difference. *p*-value < 0.0001, all values are in CFU/mL. Mean = mean value. Values are in CFU/mL.

## Data Availability

Upon reasonable request, the corresponding author M.E.M. can share any requested data.

## Abbreviations

SRP	scaling root planning
H_2_O_2_	hydrogen peroxide
W	Watt
TBC	total bacterial count
aPDT	activated photodynamic therapy
EFP	European Federation of Periodontology
AAP	American Academy of Periodontology
mm	millimeter
PCR	polymerase chain reaction
Mean	mean value
STD	standard deviation
